# Evaluation of morphine‐like effects of the mixed mu/delta agonist morphine‐6‐*O*‐sulfate in rats: Drug discrimination and physical dependence

**DOI:** 10.1002/prp2.403

**Published:** 2018-06-19

**Authors:** Jai Shankar K. Yadlapalli, Shoban Babu Bommagani, Ryan D. Mahelona, Anqi Wan, Brenda M. Gannon, Narsimha R. Penthala, Maxim Dobretsov, Peter A. Crooks, William E. Fantegrossi

**Affiliations:** ^1^ Departments of Pharmaceutical Sciences University of Arkansas for Medical Sciences Little Rock Arkansas 72205; ^2^ Department of Pharmacology and Toxicology University of Arkansas for Medical Sciences Little Rock Arkansas 72205; ^3^ Department of Anesthesiology University of Arkansas for Medical Sciences Little Rock Arkansas 72205

**Keywords:** abuse liability, delta opioid receptor (DOR), drug discrimination, mixed‐action mu/delta opioids, morphine, morphine‐6‐O‐sulfate (M6S), mu opioid receptor (MOR), physical dependence

## Abstract

Morphine‐6‐O‐sulfate (M6S) is as a mixed‐action mu/delta (μ/δ) opioid receptor agonist with high potency and analgesic efficacy. These studies used assays of drug discrimination and schedule‐controlled responding to assess abuse‐liability, tolerance, and physical dependence as compared to morphine in rats. Attempts to train 0.3 mg/kg (IP) M6S from saline failed, but all rats rapidly acquired the discrimination when the training dose was changed to 3.0 mg/kg morphine, and substitution tests showed that morphine and fentanyl both fully substituted for the training dose, M6S and M3A6S (3‐O‐acetyl ester of M6S) only partially substituted, and salvinorin A did not elicit morphine‐like effects. Tolerance to response rate‐decreasing effects was studied in rats administered either 1.0 or 3.0 mg/kg morphine or M6S before food‐reinforced operant sessions. At both unit doses, tolerance to M6S‐elicited rate suppression developed more slowly than tolerance to morphine‐induced reductions in response rates. To assess dependence, rats were maintained on 1.0 mg/kg morphine or 1.0 mg/kg M6S until food‐reinforced response rates were stable for at least 5 days. Rats were then administered saline or increasing doses of the opioid antagonist naltrexone (NTX) (0.3, 1.0, 3.0, or 10.0 mg/kg) in order to determine antagonist‐precipitated withdrawal. NTX precipitated withdrawal was similar in both morphine‐maintained and M6S‐maintained rats. In conclusion, the mixed μ/δ agonist activity of M6S failed to completely protect against the development of physical dependence, but delayed tolerance development to behavioral effects and resulted in decreased morphine‐like subjective effects, perhaps implying a decreased abuse liability over μ agonists.

**Abbreviations**
ANOVA: analysis of variance
BBB: blood brain
DMSO: dimethylsulfoxide
DOR: delta opioid receptor
FR: fixed ratio
IP: intraperitoneal
M3A6S: 3‐O‐Acetyl ester of morphine‐6‐O‐sulfate
M6S: morphine‐6‐O‐sulfate
MOR: mu opioid receptor
NTX: naltrexone
SC: subcutaneous
TO: time out

## 1 INTRODUCTION

**Figure 1:**
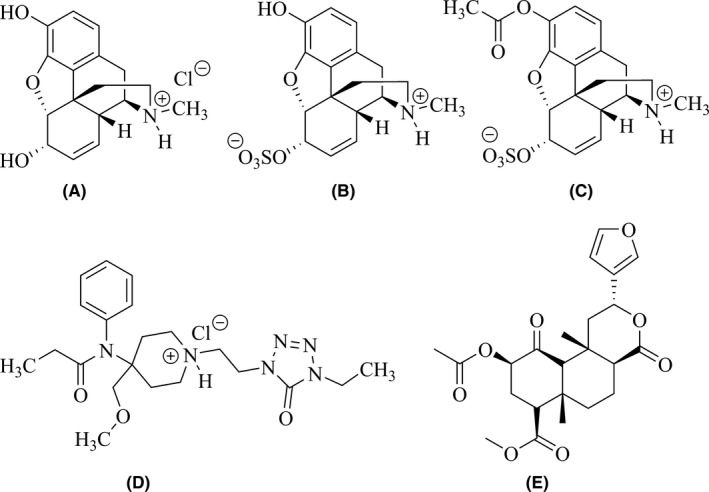
Structures of morphine hydrochloride (A), morphine‐6‐O‐sulfate (M6S; B), the 3‐O‐acetyl ester of morphine‐6‐O‐sulfate (M3A6S; C), fentanyl hydrochloride (D), and salvinorin A free base (E)

Morphine (Figure 1A) is a prototypical μ‐opioid receptor agonist used for the treatment of chronic cancer pain and for short‐term use in the treatment of strong acute pain states.^1^ Although μ‐opioid receptor agonists such as fentanyl (Figure 1D) and morphine are effective analgesics, their clinical use is limited by undesirable effects including sedation, respiratory depression, constipation, physical dependence, and high abuse liability.^2^ One strategy to improve the effectiveness and safety of μ agonist analgesics is to combine them with adjuncts that target other receptors. Toward that end, much research has been focused on development of mixed action μ/κ opioids, which are reported to have lower abuse liability than many selective μ receptor agonists.^3^, ^4^ However, a common limitation among such compounds is their typically low efficacy at both μ and κ opioid receptors,^5^, ^6^ usually resulting in low analgesic/antinociceptive efficacy in laboratory animals and in humans.^7^, ^8^, ^9^ Considering this problem, the development of opioids with mixed agonist activity at μ and δ opioid receptors has attracted attention because multiple reports suggest that (as compared to μ and δ receptor‐selective agonists) compounds activating both μ and δ receptors exhibit improved analgesic effects and safety profiles.^10^, ^11^, ^12^, ^13^

We have recently demonstrated that an O‐sulfate ester derivative of morphine, morphine‐6‐O‐sulfate (M6S) (Figure 1B), acts as a mixed μ/δ agonist in rats, attenuating acute pain in normal rats and mechanical hyperalgesia in diabetic rats with analgesic effects 3‐20‐fold more potent and 2 to 4‐fold higher in efficacy than those of morphine.^14^, ^15^ In addition, these studies also demonstrated that tolerance to M6S‐elicited analgesia develops more slowly than tolerance to morphine‐induced analgesia in each of the pain modalities tested, and that no cross‐tolerance exists between M6S and morphine 14, 15 However, the morphine‐like abuse‐related effects of M6S and its capacity to induce physical dependence have not yet been characterized.

Therefore, the goal of the present studies was to compare abuse‐related effects of M6S to those of morphine at doses within the analgesic range. We first determined the morphine‐like discriminative stimulus effects of M6S. There is a strong correlation between discriminative stimulus effects of drugs in nonverbal species and subjective effects of those same drugs as reported by humans.^16^, ^17^, ^18^ The discriminative stimulus properties of morphine and other μ opioids have been extensively investigated in several different animal species, and it has been shown that, in agreement with studies in humans, opioids generalize with one another.^19^, ^20^, ^21^ Thus, drug discrimination in laboratory rodents is a useful assay to determine the subjective effects of novel compounds in man. Then, the effects of chronic morphine or M6S on rates of food‐maintained operant responding were examined in order to determine the rate of tolerance development to this behavioral effect at doses in the analgesic range.^14^, ^15^ Finally, after complete tolerance to rate‐decreasing effects had developed, we maintained animals on daily morphine or M6S and assessed the effects of the opioid antagonist naltrexone on response rates in order to gauge antagonist‐precipitated withdrawal as a means of determining drug dependence. The use of schedule‐controlled responding to study drug tolerance, dependence and withdrawal is well‐established in behavioral pharmacology. Any drug that penetrates the central nervous system will disrupt operant responding at some dose,^22^, ^23^, ^24^ and these disruptions are readily quantifiable as decreases in response rates when compared to control conditions. Combined with the results of previous reports 14, 15 knowledge gained from this study will help understand the pharmacology of mixed μ/δ opioids and foster better strategies in the design and development of novel opioids in order to engineer a safer and more effective therapeutic for chronic pain.

### 1.1 Chemicals

Morphine hydrochloride was purchased from a commercial vendor (Merck & Co, Rahway, NJ). Morphine‐6‐O‐sulfate (M6S) as the more water‐soluble sodium salt form, and 3‐O‐acetylated morphine‐6‐O‐sulfate (M3A6S, Figure 1C) were prepared in our laboratories utilizing the synthetic route published previously.^17^ Structural confirmation for both M6S and M3A6S was accomplished using ^1^H‐ and ^13^C‐NMR spectroscopy and high‐resolution mass spectrometry. Fentanyl hydrochloride (Figure 1D) was obtained from the National Institute on Drug Abuse Drug Supply Program (Bethesda, MD) and salvinorin A (free base, Figure 1E) was synthesized according to published methods^25^ in the laboratory of Thomas Prisinzano, Ph.D. in the Department of Medicinal Chemistry at the University of Kansas School of Pharmacy (Lawrence, KS) and provided as a generous gift. Salvinorin A was first dissolved in dimethylsulfoxide (DMSO) and then dissolved in isotonic saline for injection. All other drugs were dissolved in isotonic saline. All the drugs used for behavioral studies were injected in a volume of 2 ml/kg via either intraperitoneal (IP) or subcutaneous routes (SC) of administration. Doses were calculated as those of the free bases. The doses and preinjection intervals for morphine, M6S, M3A6S, fentanyl, and salvinorin A were selected from pilot studies or from previously published reports.^14^, ^27^, ^28^, ^29^, ^30^ For M6S, a maximal dose of 5.6 mg/kg was studied since previous experiments in our lab determined this was the highest dose achievable in rodents without observing adverse sedative effects which would likely limit behavioral performance in the planned studies.^14^, ^28^ Considering the safety of the animals, the maximum doses for morphine, M6S and M3A6S were therefore limited to 5.6 mg/kg in all studies.

## 2 EXPERIMENTAL PROCEDURES

### 2.1 Subjects

Thirty‐six adult male Sprague‐Dawley rats (200‐250 g at the start of these studies; Charles River Laboratories, Wilmington, MA) were used and were pair‐housed (2 rats per cage) in individually ventilated Innovive rat cages (San Diego, CA). Six rats were used for drug discrimination experiments, and 30 rats were used for tolerance and physical dependence studies. All animals were maintained in a temperature and humidity controlled colony on a 12‐hours light/dark cycle (lights on at 0700 and off at 1900). All the rats were food restricted for the duration of all studies, and their weights were maintained at approximately 320 g with supplemental feedings after daily behavioral sessions. All behavioral studies were conducted in accordance with the Guide for the Care and Use of Laboratory Animals as adopted by the National Institutes of Health and procedures were approved by the University of Arkansas for Medical Sciences Institutional Animal Care and Use Committee (IACUC). The health of the animals was assessed daily by laboratory technicians and animal care staff.

### 2.2 Operant training

All rats were trained 5 days per week to respond in two‐lever operant conditioning boxes, using food pellets to reinforce behavior (Bio‐Serv 45 mg dustless precision pellets). During initial shaping, a single response on either lever would retract that lever and deliver a reinforcer. After a brief (10 seconds) time‐out (TO), rats were required to complete the response requirement on the remaining lever. Both levers were the reintroduced into the chamber after the TO. In this manner, rats received equivalent reinforcement from each lever, and no subsequent biases for one lever or the other were noted. Animals were initially maintained on this fixed ratio 1 (FR 1) schedule of reinforcement in sessions lasting 60 minutes or until 60 reinforcers had been earned (whichever came first.) The FR value increased by one response every 20th reinforcer earned within a given session and the FR value achieved was carried over between sessions until rats were responding under an FR 10 schedule. FR values were reset if an animal switched between levers before completion of a ratio. This segment of the training was complete when rats reached an FR 10 schedule and earned all 60 available reinforcers for at least 5 consecutive days. Rats were then randomly assigned to experimental groups.

### 2.3 Drug discrimination

#### 2.3.1 Discrimination training

**Figure 2:**
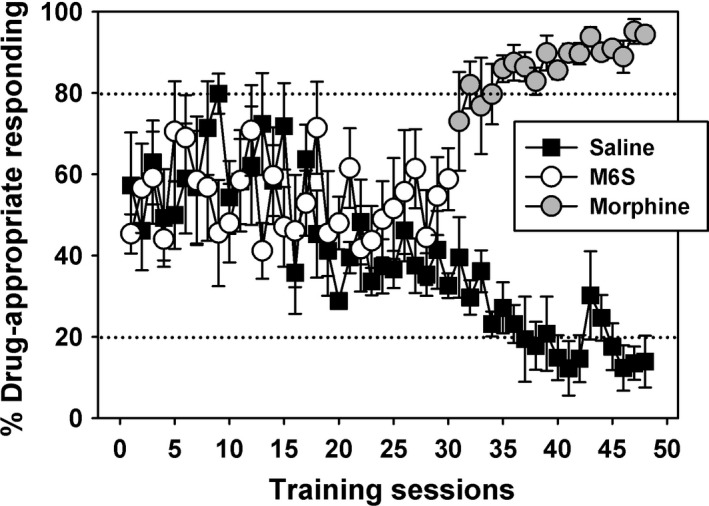
Failure of rats to attain criterion discriminative performance with 0.3 mg/kg M6S over approximately two months (~30 M6S and ~30 saline sessions), but rapid acquisition of 3.0 mg/kg morphine as a discriminative stimulus in these same subjects. *Abscissa:* percent of total responses emitted on the opioid lever. *Ordinate:* training sessions where saline, M6S or morphine was administered and discriminative performance was assessed

After initial shaping of operant responding, 6 rats were trained to discriminate 0.3 mg/kg M6S from saline after IP administration. Daily training sessions consisted of a 10‐minutes postinjection timeout period before the session began, followed by a 60‐minutes response period. During the response period, the house light and stimulus lights above each lever were illuminated and 60 food pellets were available under a FR 10 food‐maintained schedule of reinforcement. FR values were reset if an animal switched between levers before completion of a ratio. When saline was administered, completion of the response requirement on the left lever (saline‐appropriate lever) resulted in food delivery. When the training dose of M6S was administered, completion of the response requirement on the right lever (drug‐appropriate lever) resulted in food delivery. If all 60 food pellets were delivered before the end of the 60‐minute response period, the house light and stimulus lights were turned off, and the session ended. Training sessions were conducted 5 days per week. Training continued until the following three criteria were met for three consecutive sessions: (1) percentage of injection appropriate responding before delivery of the first reinforcer was >85%; (2) percentage of injection‐appropriate responding for the entire session was >90%; and (3) all available food pellets were earned during saline training sessions. These criteria were in effect for the duration of the study, such that all animals were required to pass 3 days of training prior to each substitution test. If a rat did not pass all three of the criteria previously stated, then the same training (saline or 0.3 mg/kg M6S) was repeated for the next training session. Because rats failed to acquire the discrimination of 0.3 mg/kg M6S from saline over a period of more than 2 months, animals were retrained to discriminate 3.0 mg/kg morphine from saline using the same methods as described above. This morphine discrimination was rapidly acquired by all animals (see Figure 2).

#### 2.3.2 Substitution testing

After stimulus control was established with the training drug morphine at a dose of 3.0 mg/kg, drug discrimination tests were conducted once or twice per week in each animal so long as performance did not fall below the criterion level of 80% injection‐appropriate responding in any one of the previous three training sessions. Approximately half of the test sessions were conducted the day after saline training sessions with the remainder following drug training sessions. Dose‐effect functions for morphine (0.1‐5.6 mg/kg), M6S and M3A6S (0.1‐5.6 mg/kg), fentanyl (0.01‐0.3 mg/kg), and salvinorin A (0.1‐3.0 mg/kg) were determined and ED_50_ values were calculated for all drugs. Doses for M6S and M3A6S were chosen based on studies that evaluated their analgesic effects and adverse effects. Previous research in our lab^14^, ^15^ and others^28^ have demonstrated that a dose above 5.6 mg/kg of M6S causes sedation and affects locomotor function in rats. Therefore, doses above 5.6 mg/kg of M6S and M3A6S were not tested in consideration of the safety of the animals.

For each substitution test, the distribution of responses between the two levers was expressed as a percentage of total responses emitted on the drug‐appropriate lever. Response rate was calculated for each session by dividing the total number of responses emitted on both levers by the elapsed time. Complete generalization of a training drug to a test drug is said to be present when (a) a mean of ⩾80% of all test responses occurs on the drug‐appropriate lever and (b) there is a statistically significant difference between the response distributions of the test drug and saline control sessions. An intermediate degree of generalization is defined as being present when response distributions after a test drug are <80% on the drug‐appropriate lever and are significantly different from saline control sessions. Finally, when the response distribution after a test drug is not statistically different from that in saline control sessions, an absence of generalization of the training drug to the test drug is assumed. All test sessions were conducted under extinction conditions, and failure to complete an FR 10 on either lever within 10 minutes terminated the sessions and indicated disruption of schedule‐controlled behavior. In this manner, saline, positive control drugs (morphine and fentanyl), test drugs (M6S and M3A6S) and a negative control drug (salvinorin A) were tested in all rats on at least three occasions.

### 2.4 Schedule‐controlled responding

Separate groups (n = 6 per group) of rats distinct from those used in drug discrimination studies were used in assays of food‐maintained responding designed to assess tolerance to rate‐decreasing effects of M6S and morphine and to test naltrexone‐precipitated withdrawal in rats chronically maintained on M6S or morphine. All subjects were gradually shaped to a terminal FR10 schedule using food pellet (Bio‐Serv 45 mg dustless precision pellets) reinforcement as described above. All behavioral sessions lasted 60 minutes, or until 60 reinforcers were earned.

#### 2.4.1 Tolerance to rate‐decreasing effects

Once responding was stable and reliable in two groups of rats (n = 6 per group), saline was injected SC 30 minutes before behavioral sessions, for 5 consecutive sessions. The average response rate for these five saline control sessions was set to 100% control for each individual animal in order to compensate for baseline differences in responding. In subsequent sessions, 1.0 mg/kg morphine or M6S was administered (SC) 30 minutes before behavioral sessions and these injections continued until response rates returned to 100% control levels for three consecutive sessions. Five more saline control sessions were interposed and used to recalculate 100% control response rates, and then the maintenance dose of both drugs was increased to 3.0 mg/kg. Again, these injections continued until response rates returned to 100% control levels for three consecutive sessions. Both groups of rats received morphine or M6S 7 days per week, with no exceptions.

#### 2.4.2 Naltrexone‐precipitated withdrawal

Three more groups (n = 6 per group) of rats were then shaped as previously described and maintained on saline, 1.0 mg/kg morphine or 1.0 mg/kg M6S until response rates were stable for at least 5 days. Maintenance injections were administered 30 minutes prior to behavioral sessions 7 days per week for the duration of these studies. On test days, all groups were administered saline or increasing doses of the opioid antagonist naltrexone (0.3, 1.0, 3.0, or 10.0 mg/kg) immediately before placement in operant chambers in order to indirectly assess drug dependence by determining sensitivity to antagonist‐precipitated withdrawal. Suppression of response rates following administration of naltrexone, as well as observation of behavioral signs consistent with opioid withdrawal, were used to assess severity of antagonist‐precipitated withdrawal.

### 2.5 Data analysis

Graphical presentation of all drug discrimination and physical dependence data depict mean±SEM. Drug‐discrimination data are expressed as percent drug‐appropriate responding, which is the number of responses emitted on the drug‐appropriate lever as a percentage of the total number of responses emitted. Subjects failing to emit 10 responses within 5 minutes of lever extension were deemed to be behaviorally disrupted and were not considered in the calculation of the percent drug‐appropriate responding. Generalization was said to occur if ⩾80% of the responses were on the drug‐appropriate lever, and the group mean was significantly different (*via* Kruskal–Wallis one‐way analysis of variance (ANOVA) on ranks, followed by pairwise comparisons using the Dunn's method) from saline. Nonlinear regression analysis with a variable‐slope sigmoidal dose–response curve was used to calculate the dose that elicited 50% morphine‐appropriate responding (ED_50_; with a set range of 0%‐100%) for each individual animal using Graphpad Prism 4 (La Jolla, CA). These ED_50_ values were averaged for each drug (n* *=* *6 for all drugs) to determine mean ED_50_^ ^± SEM. In the dependence and withdrawal studies, the data were expressed in terms of response rate (% control) compared to increasing doses of the opioid antagonist naltrexone to produce the dose response curve. Tolerance data were analyzed using a one‐way ANOVA and a Dunnet's post hoc test to compare the first morphine or M6S injection to all subsequent injections. The dependence and withdrawal studies were then compared across naltrexone dose and treatment groups using a two‐way ANOVA and a Bonferroni post hoc test.

## 3 RESULTS

### 3.1 Drug discrimination

Rats were initially injected with 0.3 mg/kg M6S or saline as training stimuli, however, all rats failed to achieve acceptable discriminative performance over approximately 2 months (~30 M6S [Figure 2, open circles] and ~30 saline [Figure 2, filled squares] sessions). When the training dose was changed from 0.3 mg/kg M6S to 3.0 mg/kg morphine, all subjects rapidly acquired the discrimination (Figure 2, gray circles vs. filled squares.) Thereafter, rats reliably discriminated 3.0 mg/kg morphine from saline for the remainder of these studies.

**Figure 3:**
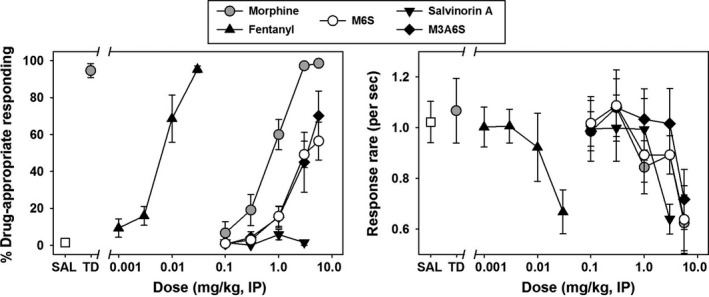
*Left panel ‐* Discriminative stimulus effects of morphine (gray circles), fentanyl (black upward triangles), M6S (white circles), salvinorin A (black downward triangles), and M3A6S (black diamonds) in rats trained to discriminate 3.0 mg/kg morphine from saline. *Abscissa:* percent of total responses emitted on the morphine lever. *Ordinate:* dose of substitution drug in mg/kg on a logarithmic scale. “SAL” refers to saline trials, while “TD” refers to administration of the training dose of morphine. *Right Panel ‐* Response rates engendered during discrimination trials by morphine and various substitution drugs. *Abscissa:* rate of lever pressing behavior, in responses per second. *Ordinate:* as described in left panel

During training sessions, rats primarily responded on the saline lever when saline was administered, and rats responded almost exclusively on the morphine lever following morphine administration. Response rates during substitution tests were generally consistent with those observed during training sessions, with saline sessions resulting in slightly higher rates than drug sessions (data not shown). When the two training stimuli were presented during substitution test sessions, saline engendered very low levels of morphine‐lever selection (Figure 3, left panel, open square), while the training dose of morphine engendered near exclusive responding on the morphine‐lever (Figure 3, left panel, gray circle.) Equivalent response rates were maintained by saline (Figure 3, right panel, open square) and the morphine training dose (Figure 3, right panel, gray circle). Substitution tests with various doses of morphine (Figure 3, left panel, gray circles) revealed dose‐dependent and full substitution for the training dose, with >80% of the total responses emitted on the drug lever at a dose of 3.0 mg/kg. Responding engendered by this dose of morphine was significantly different from the discriminative responding elicited by saline (*t* = 49.1007, *P* < .0001), and the interpolated ED_50_ for morphine was 0.847 mg/kg (95% confidence interval(CI) 0.55‐1.14 mg/kg). Morphine demonstrated a dose‐dependent decrease in response rate across the doses tested (Figure 3, right panel, gray circles).

Administration of the positive control μ opioid agonist fentanyl elicited a potent, dose‐dependent and full substitution for the morphine training dose (Figure 3, left panel, filled upward triangles), with near exclusive responding on the drug lever at a dose of 0.03 mg/kg. This highest tested dose of fentanyl elicited significantly different discriminative responding than saline (*t* = 48.28, *P* < .0001), and the interpolated ED_50_ for fentanyl was 0.007 mg/kg (95% CI: 0.004‐0.009 mg/kg). Like morphine, fentanyl dose dependently decreased response rates across the doses tested (Figure 3, right panel, black upward triangles). Substitutions with the negative control κ‐opioid agonist salvinorin A resulted primarily in responding on the saline lever, up to doses that suppressed response rates (Figure 3, left and right panels, black downward triangles). No dose of salvinorin A elicited discriminative performance significantly different from that observed following saline administration (*P* > .05).

Substitution testing with the mixed μ/δ opioid agonist M6S in the dose range of 0.1‐5.6 mg/kg elicited a dose‐dependent – but only partial – substitution for the morphine training dose. Discriminative responding elicited by the highest tested dose of M6S (5.6 mg/kg) was significantly different from that elicited by both saline and the morphine training dose (*F*(3,23) = 75.22; *P* < .0001), and the interpolated ED_50_ for M6S was 2.096 mg/kg (95% CI: 1.20‐2.99 mg/kg). Similarly, substitution testing with M3A6S, the 3‐O‐acetylated ester of the mixed μ/δ opioid agonist M6S, in the same dose range elicited a similar dose‐dependent but partial substitution for the morphine training dose as M6S. Discriminative responding elicited by the highest tested dose of M3A6S (5.6 mg/kg) was significantly different from that elicited by saline (*F* (4,29) = 18.40; *P* < .0001), however, unlike M6S, this dose of M3A6S was not statistically different from the morphine training dose (*P* > .05.). The interpolated ED_50_ for M3A6S was 2.853 mg/kg (95% CI: 1.68‐4.02 mg/kg). The interpolated ED_50_ values for both M6S and M3A6S significantly differed from that of morphine (*F* (3,23) = 18.83; *P* < .001.)

### 3.2 Tolerance to rate‐decreasing effects

**Figure 4:**
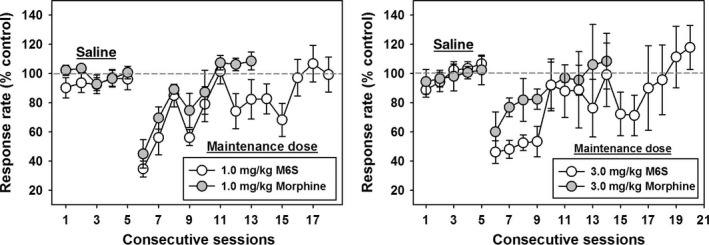
Progressive tolerance to rate‐decreasing effects of daily 1.0 mg/kg (left panel) or 3.0 mg/kg (right panel) morphine (gray circles) or M6S (white circles). *Abscissae:* rate of lever pressing behavior, expressed as percent of saline control rates. *Ordinates:* Consecutive test sessions where saline was administered on sessions 1‐5, and drug was administered thereafter

Daily administration of saline had no systematic effects on response rates during the 5 day treatment period, and the mean rate for each animal during this time was set to 100% against which to gauge rate‐decreasing effects of subsequent morphine or M6S injections (Figure 4, dotted lines in both panels). Initial administration of 1.0 mg/kg morphine (Figure 4, left panel, gray circles) or 1.0 mg/kg M6S (Figure 4, left panel, open circles) suppressed response rates by approximately 60%, but progressive tolerance to these rate‐decreasing effects developed with subsequent injections. For morphine‐maintained animals, control rates of responding were reattained after 6 injections, and once animals returned to control levels of responding, they did not deviate with successive injections (*F*(7,47) = 3.980, *P* < .05, morphine injections 6‐8 significantly different from 1st morphine injection (*P* < .05)). In contrast, rats maintained on M6S required 11 injections before stably recovering and maintaining baseline response rates (*F*(12,77) = 6.756, *P* < .05, M6S injections 6 and 11‐13 significantly different from 1st M6S injection (*P* < .05)).

When the maintenance dose of morphine or M6S was raised to 3.0 mg/kg (Figure 4, right panel), the initial injection suppressed response rates by approximately 50% in both groups, consistent with these higher maintenance doses surmounting tolerance to the lower doses. For morphine‐maintained rats, control levels of responding were again reattained within 6 injections (Figure 4, right panel, gray circles) (*F*(7,47) = 1.702, *P* < .05, morphine injections 6‐9 significantly different from 1st morphine injection (*P* < .05)). In contrast, rats maintained on 3.0 mg/kg M6S (Figure 4, right panel, open circles) required 13 injections to stably recover baseline response rates (*F*(12,77) = 3.768, *P* < .05, M6S injections 6 and 11‐13 significantly different from 1st M6S injection (*P* < .05)).

### 3.3 Naltrexone‐precipitated withdrawal

**Figure 5:**
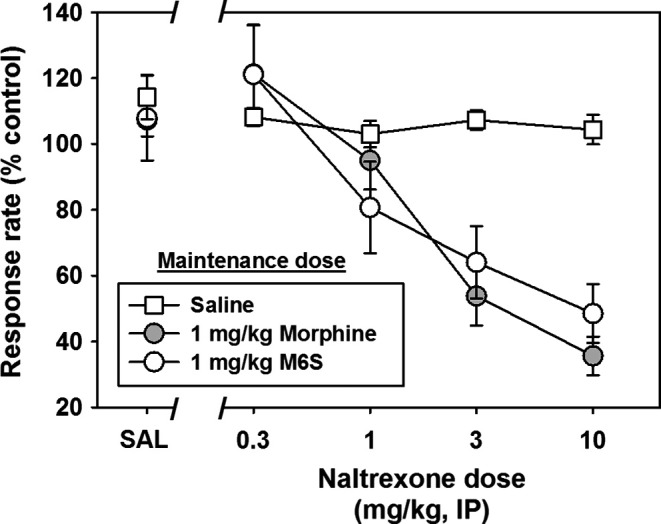
Effects of saline or various doses of naltrexone on response rates in rats maintained on daily saline (white squares), 1.0 mg/kg morphine (gray circles) or 1.0 mg/kg M6S (white circles). *Abscissa:* as described in Figure 3. *Ordinate:* dose of naltrexone in mg/kg on a logarithmic scale. “SAL” refers to saline control trials

Separate groups of rats were maintained on presession saline, 1.0 mg/kg per day morphine or M6S, as described above. In all three groups, the mean response rate for the last 3 sessions prior to administration of saline or naltrexone was normalized to 100% control. Administration of saline to rats maintained on daily saline, morphine or M6S had no systematic effects on response rates (Figure 5, points at “SAL”.) In saline‐maintained rats, presession administration of naltrexone had no significant effects on response rates up to a dose of 10.0 mg/kg (*F*(4,29) = 0.9681; *P* > .05) (Figure 5, open squares). In contrast, naltrexone dose‐dependently decreased responding in the morphine‐maintained (Figure 5, gray circles) and M6S‐maintained (Figure 5, open circles) groups, suggesting withdrawal‐related response suppressing effects in these rats consistent with postsession technician‐observed signs of opioid withdrawal, including agitation, diarrhea, wet dog shakes, and vocalization upon handling. Statistical analysis indicated significant differences among naltrexone doses (*F*(3,60) = 20.55, *P* < .001) as well as significant effects by treatment group (*F*(2,60) = 16.16, *P* < .001.) However, post hoc testing revealed that the effects of naltrexone did not differ between morphine‐ and M6S‐maintained rats (*P* > .05) at any of the naltrexone doses tested, suggesting that morphine and M6S demonstrated similar physical dependence profiles at this maintenance dose. ED_50_ values for naltrexone as a function of morphine‐ and M6S‐maintenance were not calculated because there were no significant differences in these two treatment groups.

## 4 DISCUSSION

Morphine is metabolized to two major glucuronide conjugates: morphine‐3‐O‐glucuronide (M3G) and morphine‐6‐O‐glucuronide (M6G). The major morphine metabolite (70%) in humans is M3G, which lacks any antinociceptive effects and is instead thought to antagonize the analgesic effects of morphine.^31^ On the other hand, M6G (about 10% of total metabolized morphine) is a more potent analgesic than morphine itself in both preclinical and clinical studies.^32^ While Phase 3 clinical trials with M6G have been completed, it is not clear whether further clinical trials have been initiated,^33^ and M6G is still awaiting FDA approval for use as an analgesic in humans. Indeed, controversy still surrounds the adverse effects profile of M6G, with debate continuing as to whether M6G is better or worse than morphine in this regard.^32^ Nevertheless, the analgesic efficacy of M6G clearly demonstrates that structural modification at morphine's 6‐hydroxyl position could lead to improvements in analgesic potency. This finding initially inspired us to investigate another 6‐O‐ester derivative of morphine, namely the 6‐O‐sulfate conjugate of morphine (M6S), in rat models of diabetic neuropathy.^14^, ^34^

One common adverse effect limiting the chronic use of μ opioid agonists is their notorious liability for abuse, physical dependence, and addiction.^35^ Prescription μ opioids are not only abused, but are also responsible for severe morbidity and death from overdose.^36^, ^37^ The present report provides an initial characterization of the morphine‐like discriminative stimulus effects and physical dependence properties of the novel mixed‐action μ/δ opioid M6S in rats. Here we demonstrate that, unlike morphine and fentanyl, M6S did not fully substitute for morphine in drug discrimination, although this is likely due to the limited dose range tested. Previous experiments in our lab determined adverse sedative effects at doses above 5.6 mg/kg M6S, which would likely limit behavioral performance in the planned studies,^14^, ^28^ thus, the maximum doses for morphine, M6S and M3A6S were therefore limited to 5.6 mg/kg in all studies. Nevertheless, these in vivo data suggest that the interoceptive effects of morphine are only partially recapitulated by M6S at doses that produce maximal analgesic effects.^14^ The trend of increasing morphine‐like effects as the M6S dose is increased does suggest that M6S may elicit full morphine‐like interoceptive effects at higher doses than those presently tested. However, the fact that M6S was approximately 3‐fold more potent than morphine to elicit antinociceptive effects,^15^, ^38^ but was here determined to be less potent than morphine in eliciting discriminative stimulus effects, perhaps suggests a widened therapeutic window as a function of the modification to the 6 position.

However, although M6S functioned as only a partial agonist in the drug discrimination assay across the dose range utilized, it is perhaps still reasonable to expect that M6S may possess reinforcing effects which could confer some abuse potential, although perhaps less than that observed with full μ opioid agonists such as morphine and fentanyl. Similarly, we showed that the 3‐O‐acetyl ester prodrug of M6S (M3A6S) also acted as a partial agonist in the drug discrimination study, although the maximal percent drug‐appropriate responding elicited by was not statistically different from that of morphine. M3A6S is a more lipophilic prodrug of M6S with a 400‐fold better affinity for μ‐opioid receptors than for δ‐opioid receptors.^39^ Therefore, M3A6S is a mu agonist with little to no delta opioid receptor affinity. Given the more lipophilic nature of M3A6S relative to M6S, it is plausible to propose that the greater ability of M3A6S to cross the blood brain (BBB) barrier may be one factor that might explain its apparently more robust morphine‐like discriminative profile compared to M6S. This might also highlight a more complicated process involving BBB penetration by the zwitterionic M6S molecule as a rate limiting step in μ−opioid receptor‐mediated responses such as morphine‐like discriminative stimulus effects. Towards that end, we have demonstrated the intrinsic chemical stability of M6S across various biological buffers and matrices in vitro,^40^ and these pharmacokinetic studies establish that M6S is also a stable molecule in vivo*,* with no measurable hydrolysis to morphine. Alternatively, the δ‐agonist properties of M6S may elicit effects which mask morphine‐like subjective effects, a process known as perceptual masking, which is commonly observed in discrimination studies across various species and drugs.^41^, ^42^, ^43^, ^44^ However, the μ/δ selectivity of M3A6S strongly favors affinity for μ–opioid receptors, and it also substituted only partially, which seems to argue against the perceptual masking explanation for the incomplete substitution for morphine observed with M6S and M3A6S.

Previous studies on M6S and M3A6S^45^ have demonstrated that both M6S and M3A6S are 80‐100 fold more potent than morphine in the radiant heat tail flick assay when administered directly into brain *via* intracerebroventricular injection. Therefore, because M6S only partially substituted in drug discrimination at the same dose (5.6 mg/kg) that resulted in full substitution of morphine for its training dose, it is reasonable to assume that the limited ability of M6S to cross the BBB following parenteral administration is responsible for this dramatic reduction in potency. Alternatively, the ability of M6S to activate both μ and δ opioid receptors could blunt morphine‐like interoceptive effects. Further research is clearly warranted to delineate the exact mechanism responsible for the observed partial substitution of M6S for morphine in drug discrimination. Nevertheless, given the animal welfare‐related limitations on the doses (up to 5.6 mg/kg) used in these studies for morphine, M6S and M3A6S, it remains possible that M6S and M3A6S might fully substitute for morphine at doses higher than those presently tested, although if this were the case it would still not explain the extreme loss of potency presently observed, as compared to previous studies on analgesic effects. Similarly, based on the observed trends of the present dose‐response curves, the extrapolated ED_50_ doses for M6S and M3A6S to elicit full substitution for morphine would likely fall in the range of 2.5‐3.5 mg/kg, compared to morphine's present ED_50_ value of 0.88 mg/kg. The observed ED_50_ doses of both M6S and M3A6S in the present drug discrimination study are 3‐fold higher than that of morphine and approximately 300 times higher than fentanyl, perhaps demonstrating the protective effects of μ/δ opioid agonism against morphine‐like abuse‐related effects. Furthermore, the fold difference between analgesic ED_50_ and the ED_50_ for morphine‐like discriminative effects is higher for M6S (2‐6 fold; 14, 15) compared to morphine, where the analgesic ED_50_ for morphine is either similar to or higher than the ED_50_ for morphine‐like discriminative effects, indicating that the analgesic effects of M6S are observed at doses several fold less than those which are reported as morphine‐like by rats trained to discriminate 3.0 mg/kg morphine from saline. This may suggest that analgesic effects of M6S could be obtained clinically at doses that do not elicit morphine‐like subjective effects in humans.

Often included in preclinical assessments of abuse liability,^46^ tolerance and dependence are two of several diagnostic criteria for opioid use disorder in humans. In our earlier studies,^14^, ^15^ we demonstrated that M6S induces less analgesic tolerance and lacks cross‐tolerance to morphine in both normal and diabetic rats, and our present drug discrimination study also provides evidence to suggest that M6S may have lower abuse liability than standard μ opioid analgesics. Our current study also examined the capacity of M6S to induce tolerance to rate‐decreasing effects on food‐maintained responding, and to induce physical dependence, as assessed by naltrexone precipitated withdrawal effects on response rates. First, we observed that M6S‐maintained rats (both 1.0 and 3.0 mg/kg per day) required approximately twice as many daily injections to become tolerant to rate‐decreasing effects as compared to morphine. Interestingly, these results are in close agreement with our previous in vivo studies examining tolerance to the analgesic effects of M6S and morphine.^14^, ^15^ Thus, tolerance to the behavioral effects of M6S appears to develop more slowly than with morphine, both in terms of analgesic effects and in terms of schedule‐controlled responding. But surprisingly, when challenged with naltrexone, we did not observe any differences between morphine‐maintained and M6S‐maintained rats, suggesting a similar capacity to induce physical dependence between these two opioids. Studies designed to specifically quantify observable signs of antagonist‐precipitated withdrawal following comparable dose regimens of morphine and M6S might help to better elucidate any potential differences in the topography of withdrawal from these two opioids. Although the similar withdrawal‐like naltrexone‐elicited effects on food‐maintained responding were unexpected, it remains the case that dependence on M6S may have developed more slowly with M6S than with morphine. In that regard, similar results have been observed with a mixed μ/δ opioid glycopeptide,^37^, ^47^ where it was suggested that heterodimerization of μ/δ opioid receptors (modulating neuronal network effects of μ‐ and δ‐containing neurons at a level above the individual receptor) or additional cellular regulators in neurons could modulate the induction of tolerance and dependence at the receptor level, slowing tolerance development. Indeed, it has been previously reported that drugs with affinity for μ and κ opioid receptors demonstrated different results across tests of thermal nociception, schedule‐controlled responding and drug self‐administration, and our previous and present reports with the mixed μ/δ opioid agonist M6S contributes to this growing body of literature demonstrating that novel drug‐receptor interactions may elicit distinct effects across experimental endpoints (e.g. 10, 11).

Drugs that target different binding sites can interact in diverse ways and could produce interacting effects by targeting allosteric binding sites on a common protein, binding sites on adjacent proteins physically coupled in a common receptor oligomer, physically separated proteins that modulate convergent signal cascades in a common cell, or receptors located on different cells within a common neural circuit. All of these mechanisms may contribute to different drug effects across distinct biological systems, resulting in complex or conflicting data sets across different endpoints, as may be the case presently observed with M6S. Nevertheless, despite the complexity mediating the behavioral effects of mixed μ/δ agonists and mixed μ/κ agonists, these drugs provide a strong rationale to develop new drug mixtures or inherent mixed‐action drugs because they often demonstrate a favorable side effect profile over selective agonists. Our future in vivo studies will focus on further comprehensive evaluations of the abuse‐related effects of M6S using self‐administration techniques in rodents, as well as on molecular studies dissecting cAMP tolerance and superactivation in μ/δ heteromer cells, including markers of tolerance and superactivation in primary neurons. Based on the results reported here and in conjunction with our previous studies in diabetic rats, M6S may represent a therapeutic opioid of choice due to its delayed tolerance in models of diabetic neuropathy, as well as its decreased morphine‐like discriminative stimulus effects (at doses within the analgesic range) as compared to the traditional μ opioid agonists morphine and fentanyl. However, chronic administration of M6S may still result in similar physical dependence as that observed with traditional μ opioid agonists. In conclusion, further research is recommended with M6S, as our results suggest a more complex and thus potentially interesting mechanism behind the observed differences in tolerance, dependence, and morphine‐like discriminative effects of the mixed μ/δ agonist M6S.

## DISCLOSURE

The University of Kentucky holds a patent on the compounds M6S and M3A6S described in the current work. A potential royalty stream to Dr. Peter A. Crooks may occur consistent with University of Kentucky policy. Other authors have no conflicts of interests to report.

## AUTHOR CONTRIBUTION

Participated in research design: Yadlapalli, Dobretsov, Crooks, Fantegrossi; Conducted experiments: Yadlapalli, Bommagani, Mahelona, Wan, Gannon, Fantegrossi; Contributed new reagents or analytic tools: Penthala; Performed data analysis: Yadlapalli, Fantegrossi; Contributed to the writing of the manuscript: Yadlapalli, Penthala, Crooks, Gannon, Fantegrossi.
